# Current characteristics and management of ST elevation and non-ST elevation myocardial infarction in the Tokyo metropolitan area: from the Tokyo CCU network registered cohort

**DOI:** 10.1007/s00380-015-0791-9

**Published:** 2016-01-12

**Authors:** Hideki Miyachi, Atsushi Takagi, Katsumi Miyauchi, Masao Yamasaki, Hiroyuki Tanaka, Masatomo Yoshikawa, Mike Saji, Makoto Suzuki, Takeshi Yamamoto, Wataru Shimizu, Ken Nagao, Morimasa Takayama

**Affiliations:** 1 Tokyo CCU Network Scientific Committee, Tokyo, Japan; 2 Division of Cardiovascular Intensive Care, Nippon Medical School Hospital, 1-1-5 Sendagi, Bunkyo-ku, Tokyo, 113-8603 Japan; 3 Department of Cardiovascular Medicine, Nippon Medical School, Tokyo, Japan

**Keywords:** ST elevation myocardial infarction, Non-ST elevation myocardial infarction, In-hospital mortality, Risk factors

## Abstract

Limited data exists on ST-segment elevation myocardial infarction (STEMI) and non-STEMI (NSTEMI) managed by a well-organized cardiac care network in a metropolitan area. We analyzed the Tokyo CCU network database in 2009–2010. Of 4329 acute myocardial infarction (AMI) patients including STEMI (*n* = 3202) and NSTEMI (*n* = 1127), percutaneous coronary intervention (PCI) was performed in 88.8 % of STEMI and 70.4 % of NSTEMI patients. Mean onset-to-door and door-to-balloon times in STEMI patients were shorter than those in NSTEMI patients (167 vs 233 and 60 vs 145 min, respectively, *p* < 0.001). Coronary artery bypass graft surgery was performed in 4.2 % of STEMI and 11.4 % of NSTEMI patients. In-hospital mortality was significantly higher in STEMI patients than NSTEMI patients (7.7 vs 5.1 %, *p* < 0.007). Independent correlates of in-hospital mortality were advanced age, low blood pressure, and high Killip classification, statin-treated dyslipidemia and PCI within 24 h were favorable predictors for STEMI. High Killip classification, high heart rate, and hemodialysis were significant predictors of in-hospital mortality, whereas statin-treated dyslipidemia was the only favorable predictor for NSTEMI. In conclusion, patients with MI received PCI frequently (83.5 %) and promptly (door-to-balloon time; 66 min), and had favorable in-hospital prognosis (in-hospital mortality; 7.0 %). In addition to traditional predictors of in-hospital death, statin-treated dyslipidemia was a favorable predictor of in-hospital mortality for STEMI and NSTEMI patients, whereas hemodialysis was the strongest predictor for NSTEMI patients.

## Introduction

Acute myocardial infarction (AMI) is a major cause of morbidity and mortality worldwide. The direct relationship between higher survival rates of ST elevation myocardial infarction (STEMI) patients and the earlier primary PCI can be performed has been well established [1, 2]. Current updated guidelines for non-STEMI (NSTEMI) in Japan, US, and Europe recommend an early invasive strategy within 24-h for high-risk patients defined as a GRACE score of >140 [3, 4]. However, little data regarding NSTEMI time courses is available in Japan.

The Tokyo CCU network is a well-organized cardiac care network. All 67 hospitals participating in the Tokyo CCU network have as a goal to perform emergency PCI within 1 h from arrival at the hospital. Deducing from the regional distribution of these 67 hospitals, the Tokyo CCU network probably includes 95 % of all patients who had an AMI within the greater Tokyo area, moreover there are 13 million people in the Tokyo metropolitan area. Furthermore, gathering data from this cardiac care network on the current practices and in-hospital survival rates is vital to better serve the highly populated Tokyo metropolitan area.

The aim of the present study was to clarify current characteristics, managements, time courses, and in-hospital outcomes in STEMI and NSTEMI patients in the Tokyo CCU network registered cohort within the contemporary real-world setting.

## Methods

### The Tokyo CCU network registry

**Fig. 1:**
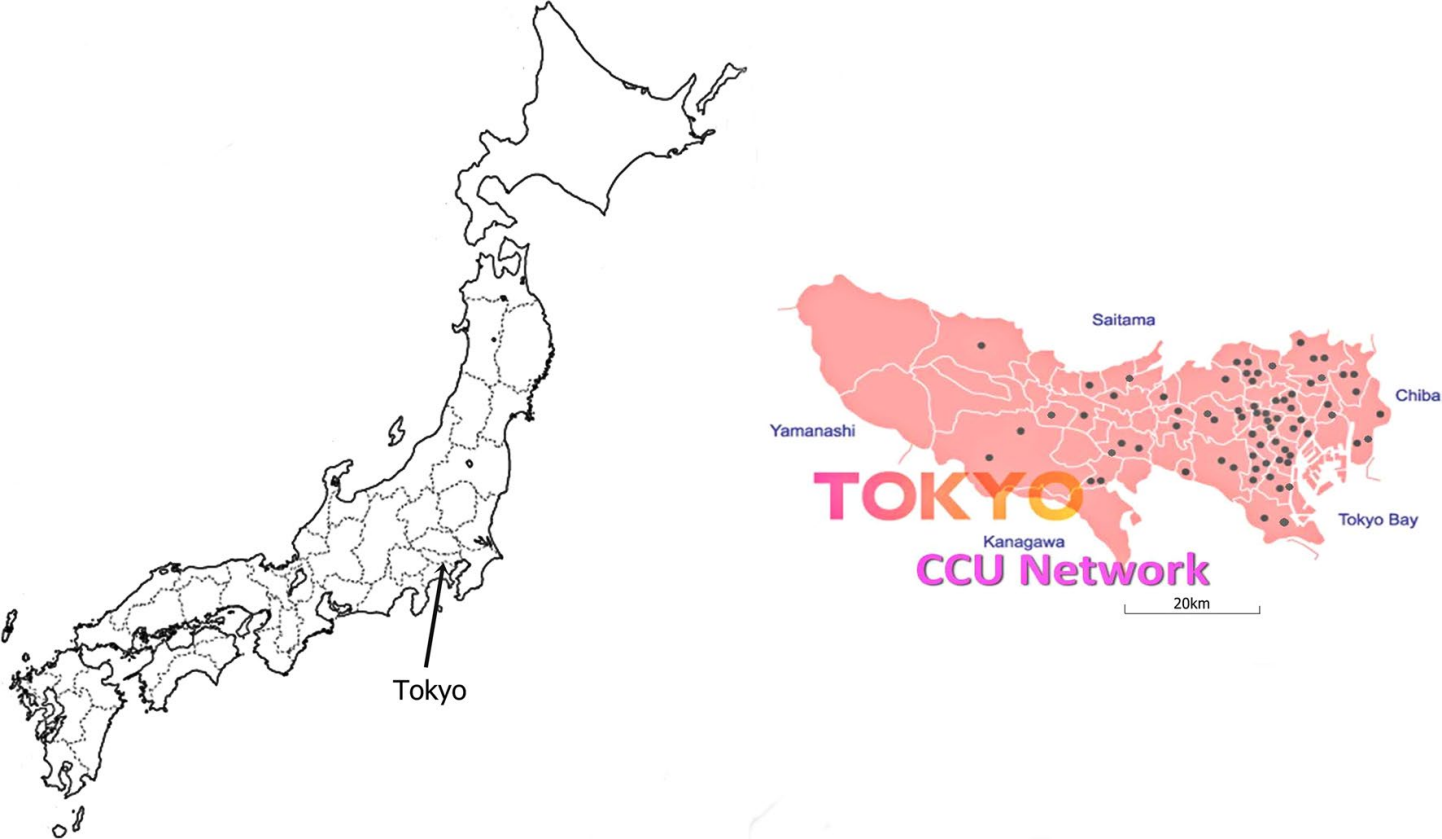
Japanese and Tokyo map. This map shows the regional distribution of 67 hospitals participating in Tokyo CCU network

The Tokyo CCU network was established in 1978 with the goal to treats patients with emergency cardiovascular events as promptly as possible, with the help of ambulance units dispatched through the Tokyo Fire Department [5]. The Tokyo CCU network database is an continually ongoing multicenter registry that prospectively collects information regarding emergency admissions to acute care facilities [6, 7]. The Tokyo CCU network was originally comprised of 12 major participating hospitals in 1978. Today it is comprised of 67 hospitals, serving a population of 13 million in the Tokyo metropolitan area (Fig. 1). Because all data is anonymously catalogued, informed consents are not required. In accordance with rules set forth by the Ministry of Internal Affairs and Communications, patient data collection policies are displayed at the emergency entrance of each individual Tokyo CCU network institution. The registry’s purpose and mission are displayed on pamphlets, which also explain CCU personal information protections to emergency admission patients and their families. Data collection was performed via individual chart review by trained data collection personnel. The quality of submitted data is maintained through annual data quality checks which include point-of-entry mechanistic controls. No extramural funding was used to support this work. Each Tokyo CCU network hospital is accredited by the Metropolitan Tokyo Government and participates in the Tokyo citywide system of acute cardiac care (acute myocardial infarction, unstable angina, arrhythmia, acute heart failure, aortic dissection, and pulmonary embolism).

### Patients and data collection

This study was performed using Tokyo CCU network registered cohort data collected from January 2009 to December 2010. Only the data of patients with AMI were selected. Clinical diagnoses of STEMI and NSTEMI, based on the 2007 universal definition, was made by individual cardiologists at Tokyo CCU network institutions [8]. All patients were prospectively registered at admission. For this study, STEMI and NSTEMI were redefined as the follow; STEMI, patients must have had chest symptoms, ST-segment elevation in 2 contiguous leads or left bundle branch block, and an elevated biochemical marker of myocardial necrosis (troponin T >0.1 ng/ml or creatine phosphokinase twofold the upper limit of normal); NSTEMI, patients must have had chest symptoms, ST-segment depression or T-wave inversion in 2 contiguous leads, and an elevated biochemical marker of myocardial necrosis. AMI patients that did not fulfill either definition in present study were excluded. The therapeutic strategies of STEMI and NSTEMI depended on the practice of each individual institution and individual cardiologist. However, all patients were treated based on guidelines set forth by the Japanese Circulation Society and ACC/AHA for the diagnosis and treatment of AMI [2, 3].

Clinical course information, as well as the diagnostic and therapeutic management of patients entering the registry, was obtained by means of a standardized questionnaire made by the steering committee. The following data was collected on patients with STEMI and NSTEMI: (1) time courses from onset of symptoms to admission to cardiovascular care unit and coronary revascularization; (2) patients’ demographics, presenting characteristics, personal and/or family history of cardiovascular disease, and cardiovascular risk factors; (3) cardiovascular treatments during the hospitalization; (4) in-hospital all-cause death. Completed case report forms were checked by the Tokyo CCU network scientific committee for the validity and consistency of the data compiled.

For this study, 5291 consecutive patients were identified that satisfied STEMI and NSTEMI definition. However, data from 962 patients was incomplete and those cases were excluded from the analysis. Therefore data from the remaining 4329 patients were analyzed.

### Statistical analysis

Categorical variables were tested using the Chi-square test or, if not applicable, Fisher’s exact test. Continuous variables are presented as means and standard deviations and were tested using the Student’s *t* test or the Mann–Whitney *U* test. To identify predictors of in-hospital mortality, univariate and multivariate logistic regression analyses were performed with 3 models: model 1 was derived from the overall cohort, model 2 was derived from STEMI patients, and model 3 was derived from NSTEMI patients. Selected variables were advanced age, male gender, systolic blood pressure, heart rate, Killip classification, percutaneous coronary intervention (PCI) of 24 h from onset, smoking, diabetes mellitus, dyslipidemia treated with statin, hyperuricemia, past MI, history of heart failure (HF), old cerebral infarction (CI), hemodialysis, and history of PCI. Only variables with a *p* value ≤0.05 identified with univariate analysis were entered as explanatory variables in subsequent multivariate models. All probability values were 2 tailed, and values of *p* < 0.05 were considered statistically significant. Statistical analysis was performed using IBM SPSS statistics 20 (IBM corporation, Armonk, NY, USA).

## Results

### Patients’ demographics

Clinical patient characteristics are summarized in Table 1. The average age was 68 years old, and 74.6 % were male. Approximately, two-thirds had hypertension, 40 % dyslipidemia, one-third diabetes mellitus, and one-third were current smokers. With regards to location of onset, 67.8 % of patients experienced their first attack at home, 22 % away from home, 5.9 % in a hospital, and 4.2 % were unknown.

**Table 1:** Patients’ clinical characteristics

	Total (*n* = 4329)	STEMI (*n* = 3202)	NSTEMI (*n* = 1127)	*p* value
Age (years)	67.9 ± 13.0	67.3 ± 13.1	69.5 ± 12.5	<0.001
Male (%)	74.6	75.5	72.3	0.041
BMI (kg/m^2^)	23.7 ± 4.0	23.7 ± 3.8	23.8 ± 4.5	0.203
Systolic BP (mmHg)	134.6 ± 36.7	133.1 ± 40.6	140.3 ± 29.4	<0.001
HR (beat/min)	79.5 ± 21.2	79.0 ± 21.8	81.0 ± 19.5	0.014
Cardiovascular history				
MI (%)	9.2	7.3	13.7	<0.001
PCI (%)	8.1	6.6	11.7	<0.001
CABG surgery (%)	1.9	1.0	4.0	<0.001
Heart failure (%)	1.7	1.3	2.6	0.017
Cerebral infarction (%)	5.7	5.3	6.7	0.118
Hemodialysis (%)	3.9	2.1	8.0	<0.001
PAD (%)	1.5	1.3	2.2	0.064
Coronary risk factor				
Hypertension (%)	61.9	60.9	64.4	0.059
Dyslipidemia (%)	43.4	43.5	43.1	0.847
Diabetes mellitus (%)	32.5	31.1	36.0	0.006
Current smoker (%)	32.9	34.3	29.6	0.009
Hyperuricemia (%)	5.1	5.1	5.3	0.795
Location of onset				
Home	67.8	66.8	70.2	0.527
Outside the house	22.0	24.1	17.2	
In hospital	5.9	5.2	7.7	
Unknown	4.2	3.9	4.9	
Killip classification				
Class 1 (%)	74.6	74.3	75.6	0.402
Class 2 (%)	13.4	13.2	14.0	
Class 3 (%)	5.4	4.7	7.1	
Class 4 (%)	6.5	7.8	3.3	
Time course				
Onset-to-Door	(*n* = 3266)	(*n* = 2554)	(*n* = 714)	
Median time (min)	180	165	233	<0.001
Interquartile range (min)	80–475	74–423	110–653	
Door-to-Ballon time	(*n* = 2401)	(*n* = 1969)	(*n* = 432)	
Median time (min)	66	60	145	<0.001
Interquartile range (min)	39–130	35–104	61–681	

*STEMI* ST-segment elevation myocardial infarction, *NSTEMI* non-ST-segment elevation myocardial infarction, *BMI* body mass index, *BP* blood pressure, *HR* heart rate, *MI* myocardial infarction, *PCI* percutaneous coronary intervention, *CABG* coronary artery bypass graft, *PAD* peripheral artery disease

When comparing subgroups, NSTEMI patients were typically older than those with STEMI. Moreover, NSTEMI patients were more likely to have history of cardiovascular disease and additional coronary risk factors with the exception to hemodialysis patients, those with dyslipidemia, and/or are current smokers. The distribution of Killip classification and location of onset were statistically similar between the 2 groups. Of the patients with PCI for which time course information was available (*n* = 3266), the median onset-to-door time (the median time interval between the onset of symptom and the patient’s hospital arrival) was 180 min. The interval was longer in NSTEMI patients (*n* = 714) than in STEMI patients (*n* = 2554) (median time was 233 vs 165 min; *p* < 0.001). The median door-to-balloon time (the median time interval between the patient’s arrival at the hospital and flow restoration by means of balloon inflation) was 60 and 145 min in STEMI (*n* = 1969) and NSTEMI (*n* = 432), respectively. Of STEMI patients (*n* = 1969), 68.2 % (*n* = 1343) and 90.5 % (*n* = 1784) underwent primary PCI within 90 min and 6 h upon arrival to the hospital, respectively. The appropriateness of primary PCI was analyzed based on US guideline of appropriate use criteria for coronary revascularization [9]. Of STEMI patients, to which primary PCI was available to onset-to-balloon time (*n* = 1473), 89.4 % of these patients (*n* = 1318) received primary PCI within 12 h from onset of symptom.

### Management and outcome

Table 2 shows the use of pharmacological therapies during hospitalization. Heparin was administered as an anticoagulant therapy to 87.9 % of patients, whereas, aspirin was prescribed as antiplatelet therapy to 93.6 %. Eighty percent of patients who underwent PCI received dual antiplatelet therapy (DAPT) during their admission. Angiotensin-converting enzyme inhibitor (ACEI)/angiotensin II receptor blocker (ARB), β blocker, and calcium antagonists were prescribed to approximately 60, 40, and 20 % of all patients, respectively. Statin was administered to 65 % of all patients. In-hospital pharmacological therapies were used more frequently in STEMI patients than NSTEMI patient, with the exception of calcium antagonists, where its use was higher in NSTEMI. In STEMI patients, the proportion of thrombolytic therapy including facilitated PCI and thrombolysis alone was just 3.1 % (data not shown).

**Table 2:** Pharmacological therapies during hospitalization

	Total (*n* = 3241)	STEMI (*n* = 2221)	NSTEMI (*n* = 1020)	*p* value
Heparin (%)	87.9	88.8	85.8	0.015
Aspirin (%)	93.6	94.6	91.3	<0.001
Thienopyridine (%)	73.1	77.4	63.6	<0.001
DAPT	71.7	76.2	62.0	<0.001
PCI with DAPT	80.7	82.1	76.6	0.002
Statin (%)	65.0	68.6	57.2	<0.001
ACEI/ARB (%)	58.3	61.5	51.4	<0.001
Nitrate (%)	44.2	44.8	42.9	0.32
β blocker (%)	38.8	41.7	32.5	<0.001
Nicorandil (%)	41.5	46.0	31.7	<0.001
Diuretic (%)	23.2	23.5	22.6	0.62
Calcium antagonist (%)	17.6	15.2	22.7	<0.001
Oral antidiabetic drug (%)	9.8	10.1	9.1	0.41
Insulin (%)	6.5	6.2	7.4	0.22

*DAPT* dual antiplatelet therapy, *ACEI* angiotensin-converting enzyme inhibitor, *ARB* angiotensin receptor blocker

Angiographic findings, invasive procedures, time courses, and in-hospital deaths are shown in Table 3. Most patients (92.2 %) underwent coronary angiography. Culprit lesions included the right coronary artery and the left anterior descending artery in a majority of STEMI patients, whereas culprit lesions involved the left main trunk, left circumflex artery, and undefined lesions appeared more frequently in NSTEMI patients than STEMI patients. Of NSTEMI patients, 55.2 % had multi vessel diseases, and almost the same proportion of STEMI patients (55.4 %) had single vessel disease. With respect to invasive procedures, 83.5 % of all patients underwent PCI during hospitalization; STEMI and NSTEMI patients accounted for 88.8 and 70.4 % of all patients, respectively (*p* < 0.001). Of all patients who underwent PCI, bare metal stents and drug eluting stents were inserted in 60.0 and 27.0 %, respectively. Bypass surgery was performed more frequently in NSTEMI patients than STEMI patients (11.4 vs. 4.2 %, respectively, *p* < 0.001).

**Table 3:** Angiographic findings, invasive procedures, and in-hospital outcome

	Total (*n* = 4329)	STEMI (*n* = 3202)	NSTEMI (*n* = 1127)	*p* value
Angiographic findings				
CAG (%)	92.2	94.1	87.7	<0.001
Culprit lesion				<0.001
RCA	34.5	38.7	23.4	
LMT	3.3	2.6	5.2	
LAD	45.2	47.8	37.9	
LCX	13.9	9.7	25.2	
Others	0.5	0.2	1.3	
Undefined	2.6	0.9	6.9	
The number of diseased vessels				<0.001
Single vessel	52.9	55.4	44.8	
Multi vessel	47.1	44.6	55.2	
Treatment				
PCI (%)	83.5	88.8	70.4	<0.001
PCI within 24 h (%)	73.2	81.1	53.7	<0.001
Thrombus Aspiration (%)	55.1	61.9	28.3	<0.001
Distal protection device (%)	7.5	8.1	4.8	0.006
The type of stent				<0.001
BMS	60.0	64.2	46.6	
DES	27.0	22.4	41.7	
Unknown	13.0	13.4	11.7	
CABG (%)	6.3	4.2	11.4	<0.001
IABP	12.2	11.5	14.0	0.039
PCPS	3.6	4.1	1.9	<0.001
Outcome				
In-hospital mortality (%)	7.0	7.7	5.1	0.007

*BMS* bare metal stent, *DES* drug eluting stent

STEMI patients had significantly a higher in-hospital mortality rate than NSTEMI patients (7.5 vs. 5.1 %, *p* = 0.007) (Table 4).

**Table 4:** Univariate and multivariate logistic regression analysis

AMI patients (*n* = 2724)				
	Univariate		Multivariate	
	Odd ratio (95 % CI)	*p* value	Odd ratio (95 % CI)	*p* value
Dependent variable; in-hospital mortality				
Independent variable				
Age (per 1-year increase)	1.056 (1.045–1.067(	<0.001	**1.048 (1.030**–**1.066)**	<0.001
Male	0.631 (0.492–0.809)	<0.001	0.744 (0.499–1.110)	0.147
Systolic BP (per 10 mmHg increase)	0.982 (0.978–0.987)	<0.001	**0.993 (0.987**–**0.998)**	0.011
HR (per decile)	1.173 (1.115–1.235)	<0.001	**1.073 (1.009**–**1.142)**	0.025
STEMI	1.555 (1.156–2.092)	0.004	1.582 (0.980–2.558)	0.060
Killip	3.019 (2.713–3.359)	<0.001	**2.515 (2.155**–**2.936)**	<0.001
PCI within 24 h	0.462 (0.363–0.589)	<0.001	1.073 (1.009–1.142)	0.144
Smoking	0.424 (0.309–0.582)	<0.001	0.954 (0.613–1.482)	0.833
Diabetes mellitus	1.243 (0.961–1.609)	0.097		
Dyslipidemia treated with statin (reference; no dyslipidemia)	0.236 (0.161–0.346)	<0.001	**0.365 (0.223**–**0.596)**	0.045
Hyperuricemia	1.240 (0.732–2.101)	0.424		
Prior MI	1.805 (1.256–2.594)	0.001	1.447 (0.818–2.560)	0.205
History of heart failure	2.769 (1.432–5.355)	0.002	0.994 (0.405–2.445)	0.990
Old cerebral infarction	2.045 (1.347–3.104)	0.001	1.169 (0.631–2.168)	0.619
Hemodialysis	2.496 (1.542–4.040)	<0.001	**2.294 (1.107**–**4.753)**	0.026
History of PCI	1.507 (1.028–2.209)	0.036	1.346 (0.743–2.445)	0.327

Statistical significant values (*p* < 0.05) in multivariate analysis are highlighted in bold

### Multivariate analysis

After logistic regression analysis, independent prognosticators of in-hospital mortality in STEMI patients were: advanced age, low blood pressure, and high Killip classification; whereas statin-treated dyslipidemia and PCI performed within 24 h from onset were associated lower risk of death (Table 5). For NSTEMI patients, independent prognosticators of in-hospital mortality were: high heart rate, high Killip classification, and hemodialysis (Table 6); whereas statin-treated dyslipidemia was the only factor associated with lowering risk of death. Independent predictors of in-hospital mortality in AMI patients were very comparable to prognosticators for STEMI and NSTEMI (Table 4). Killip classification was the strongest predictor of in-hospital mortality in all groups. Since statin-treated dyslipidemia was associated with lowering risk of death, we compared patients with statin-treated dyslipidemia to patients without dyslipidemia. STEMI patient with statin-treated dyslipidemia had more Killip 1 than patients without dyslipidemia, however, this was not the case in NSTEMI patients. More frequently than patients without dyslipidemia, both STEMI and NSTEMI patients with stain-treated dyslipidemia received PCI within 24 h. Door-to-balloon time was similar between cases with stain-treated dyslipidemia and without dyslipidemia in both STEMI and NSTEMI patients. Excluding history of cerebral infarction, the history of cardiovascular disease and coronary risk factors were similar among cases with stain-treated dyslipidemia and without dyslipidemia in STEMI and NSTEMI patients. Cases without dyslipidemia had more history of cerebral infarction than cases with stain-treated dyslipidemia in both STEMI and NSTEMI patients groups (data not shown).

**Table 5:** Univariate and multivariate logistic regression analysis

STEMI patients (*n* = 2133)				
	Univariate		Multivariate	
	Odd ratio (95 % CI)	*p* value	Odd ratio (95 % CI)	*p* value
Dependent variable; in-hospital mortality				
Independent variable				
Age (per 1-year increase)	1.069 (1.055–1.081)	<0.001	**1.058 (1.037**–**1.079)**	**<0.001**
Male	0.506 (0.385–0.665)	<0.001	0.703 (0.450–1.098)	0.121
Systolic BP (per 10 mmHg increase)	0.773 (0.724–0.826)	<0.001	**0.865 (0.792**–**0.946)**	**<0.001**
HR (per decile)	1.157 (1.095–1.222)	<0.001	1.046 (0.978–1.119)	0.190
Killip	3.105 (2.757–3.498)	<0.001	**2.570 (2.167**–**3.049)**	**<0.001**
PCI within 24 h	0.335 (0.254–0.441)	<0.001	**0.625 (0.406**–**0.962)**	**0.033**
Smoking	0.370 (0.258–0.529)	<0.001	0.913 (0.547–1.523)	0.727
Diabetes mellitus	1.156 (0.865–1.546)	0.327	–	
Dyslipidemia treated with statin (reference; no dyslipidemia)	0.198 (0.127–0.310)	<0.001	**0.376 (0.217**–**0.651)**	**<0.001**
Hyperuricemia	1.347 (0.777–2.430)	0.274	–	
Prior MI	1.508 (0.947–2.400)	0.084	–	
History of heart failure	3.044 (1.389–6.673)	0.005	0.917 (0.322–2.616)	0.872
Old cerebral infarction	2.091 (1.303–3.355)	0.002	1.061 (0.531–2.120)	0.867
Hemodialysis	2.374 (1.190–4.734)	0.014	1.610 (0.583–4.448)	0.359
History of PCI	1.407 (0.876–2.259)	0.157	–	

Statistical significant values (*p* < 0.05) in multivariate analysis are highlighted in bold

**Table 6:** Univariate and multivariate logistic regression analysis

NSTEMI patients (*n* = 646)				
	Univariate		Multivariate	
	Odd ratio (95 % CI)	*p* value	Odd ratio (95 % CI)	*p* value
Dependent variable; in-hospital mortality				
Independent variable				
Age (per 1-year increase)	1.015 (0.993–1.038)	0.182	–	
Male	1.637 (0.837–3.203)	0.150	–	
Systolic BP (per 10 mmHg increase)	0.973 (0.850–1.113)	0.688	–	
HR (per decile)	1.290 (1.123–1.482)	<0.001	**1.165 (1.012**–**1.342)**	**0.034**
Killip	2.617 (2.045–3.348)	<0.001	**2.420 (1.727**–**3.392)**	**<0.001**
PCI within 24 h	0.728 (0.416–1.274)	0.267	–	
Smoking	0.695 (0.349–1.383)	0.300	–	
Diabetes mellitus	1.833 (1.025–3.280)	0.041	1.205 (0.550–2.641)	0.641
Dyslipidemia treated with statin (reference; no dyslipidemia)	0.415 (0.194–0.887)	0.023	**0.368 (0.139**–**0.977)**	**0.045**
Hyperuricemia	0.771 (0.182–3.270)	0.724	–	
Prior MI	3.390 (1.805–6.368)	<0.001	1.691 (0.638–4.485)	1.691
History of heart failure	2.715 (0.783–9.410)	0.115	–	
Old cerebral infarction	2.080 (0.850–5.091)	0.109	–	
Hemodialysis	3.838 (1.872–7.870)	<0.001	**3.627 (1.326**–**9.920)**	**0.012**
History of PCI	2.217 (1.122–4.379)	0.022	1.582 (0.585–4.275)	0.366

Statistical significant values (*p* < 0.05) in multivariate analysis are highlighted in bold

## Discussion

To the best of our knowledge, the Tokyo CCU network is the largest citywide, most well-organized cardiac care system for a metropolitan city area in the world. This citywide, multicenter, prospective observational registry provides insight on the characteristics, management, and in-hospital mortality rates of both STEMI and NSTEMI patients. NSTEMI patients tended to have more extensive medical histories, including more cardiovascular events and coronary risk factors, than STEMI patients. Nevertheless, NSTEMI patients tended to receive in-hospital pharmacological therapies and undergo PCI less frequently than STEMI patients. These tendencies are not unique to Tokyo CCU network and are similar to many registries in Japan and other countries [10–12].

For this study, we focused on the comparing in-hospital mortality with previous studies performed in other countries. In the other studies, the in-hospital mortality rate was 4.6–8.9 % in STEMI and 4.2–5.8 % in NSTEMI [11–14], thus our results are comparable to other various large and/or small-scale registries. However, the approach to the management of coronary revascularization in Japan is unique and comparisons made between the Tokyo CCU network and foreign registries are unreliable. Therefore, we compared our data with the most notable registry in Japan, namely, the Prevention of AtherothrombotiC Incidents Following Ischemic Coronary attack (PACIFIC) registry. The PACIFIC registry is comprised of 96 Japanese regional core hospitals, that have the facilities for advanced interventional therapy, participating in a large-scale, prospective observational study [10]. In the PACIFIC registry, in-hospital mortality for STEMI and non-ST elevation acute coronary syndrome (which includes NSTEMI and unstable angina) was 4.1 and 1.3 %, respectively. The PACIFIC registry data was characterized by a high proportion of patients of who received PCI (93.5 %) and low proportions of Killip class 3 and 4 (2.9 and 2.8 %, respectively), which contrasts with the present study. Whereas, all participating PACIFIC registry hospitals were regional core hospitals and had facilities for advanced interventional therapy, Tokyo CCU network hospitals have varying degrees of different capabilities. Roughly 30 % of Tokyo CCU network hospitals have fewer than 4 beds in the CCU, are incapable of performing emergency cardiovascular surgery, and come across less than 200 PCIs annually. Generally, to perform PCI, many Japanese hospitals with a cardiology unit have their own catheterization laboratories. Accordingly, primary PCI is performed in what may be considered relatively small-scale hospitals. In terms of hospital size and capability, we believe that our registry reflects more closely the real-world clinical data of a Japanese metropolitan area. Of particular note, there was a higher proportion of NSTEMI patients with hemodialysis (8.0 %) for this study. It is known that Japan has one of the highest hemodialysis rates when compared with elsewhere in the world [15, 16], and correspondingly patients with chronic kidney disease on hemodialysis have higher rates of cardiovascular morbidity and mortality [17].

With regards to onset-to-door times, there are two main components that may potentially cause delays; the pre-hospital patient decision delay and pre-hospital transportation delay [18]. According to several studies about patient delay in Europe and the US, it remains a controversial topic whether education campaigns significantly contribute to delay reductions from onset to hospital arrival [19–21]. Furthermore, the pre-hospital transportation delay depends largely on the emergency medical service response time in Tokyo. The median time interval from first medical contact to hospital presentation was 37 min in the Tokyo CCU network (data not shown as table). This time interval is acceptable, but streamlining the process of hospital selection may be needed.

With regards to pharmacological therapies, β blocker was prescribed to fewer patients in comparison to the French OPERA registry [12]. However, β blocker utilization rates in Japan are generally lower than that of Western countries, even though favorable β blocker effects on AMI have been reported [22–25]. The plausible explanation for lower β blocker utilization is that the cardiovascular event rate after AMI for Japanese patients is lower than that of Western patients and coronary spasm incidences for Japanese patients is higher than that of Western patients [26]. Nicorandil utilization rates were extremely high in this study (44.2 %). The J-wind study in Japan showed that nicorandil had a favorable effect for acute MI patients and improved the left ventricular ejection fraction even though it did not reduce infarct size or the incidence of cardiac death [27]. Consequently, there factors may affect the selection of medications.

Multivariate logistic regression analysis showed that statin-treated dyslipidemia was associated with lower risk in both STEMI and NSTEMI patients. These findings lend further support to the effectiveness of statin treatment in STEMI and NSTEMI [28, 29]. Additionally, PCI performed within 24-h was associated with lower in-hospital mortality rates in STEMI patients. It is well established that primary PCI is greatly beneficial for STEMI [1, 2]. In contrast, the optimal intervention time for the treatment of NSTEMI patients has been debated for years [30–32]. Several randomized trials have shown that in NSTEMI patients, an early-intervention strategy was not superior to a delayed-intervention strategy for the prevention of death [31, 32]. On the other hand, recent updated guidelines for NSTEMI recommended an early invasive strategy within 24-h for high-risk patients defined as GRACE score of >140 [3, 4]. In this study’s NSTEMI data, there was no statistical superiority between survival rates for PCI performed within 24-h versus PCI performed after 24 h. A reason for our findings in multivariate logistic regression is the inclusion of all patients, but not selected patients.

These study findings have clinical implications and can provide much information to cardiologists. At the level of statistical significance, the second strongest predictor of in-hospital mortality was hemodialysis in NSTEMI patients. Therefore, cardiologists and nephrologists must pay greater attention in cases involving the cardiovascular events of patients with hemodialysis.

Furthermore, current STEMI guidelines recommend a door-to-balloon time of 90 min or less for patients undergoing primary PCI [2, 33]. Door-to-balloon time in this study was shorter than that of recommended guidelines, and approximately 70 % of patients were prepared to undergo primary PCI within 90 min upon arrival to the hospital. Moreover, according to US guidelines of appropriate use criteria [9], coronary revascularization is appropriate 12 h or less from the onset of symptoms for STEMI patients. For this study, 89.4 % of patients were appropriately eligible to receive primary PCI based on US guideline of appropriate use criteria for coronary revascularization. Thus the promptness and appropriateness of primary PCI were within acceptable parameters. Nevertheless, in-hospital mortality in this study was not particularly low. Approaches with the potential to improve in-hospital mortality may include reducing the number of patients with door-to-balloon times greater than 90 min, increasing the patients’ awareness of symptoms, and shorting the transfer time between first medical contact and hospital presentation.

## Limitations

The present study is subject to some intrinsic limitations associated with an observational study. Patients that did not need to be hospitalized in the CCU were not registered in this study, therefore, low-risk patients may have been excluded. Since we redefined the STEMI and NSTEMI as troponin T >0.1 ng/ml in this study, there were some unavailable and missing data for each patient existed. Of 962 patients who were excluded, 63.5 % were not described as having STEMI or NSTEMI. A proportion of excluded patients underwent PCI within 24 h upon arrival to the hospital were lower than those of study patients (68.8 vs. 73.2 %, *p* = 0.06). The distribution of Killip class for excluded patients was markedly different from those of study patients (1, 69.0 %; 2, 13.4 %; 3, 7.6 %; 4, 10.0 %; *p* = 0.010). In-hospital mortality rate of excluded patients was higher than that of study patients (10.9 vs. 7.0 %, *p* < 0.001). Therefore, severe patient cases may have been excluded, and selection bias may have affected the results. Moreover, the number of registered patients varied widely among hospitals. Thus, large institutional variations may have occurred. In addition, important pieces of information such as long-term outcomes and events, left ventricular ejection fraction (LVEF), medication doses, and the initiation times of medications were not assessed in the present study. Within the present study, we showed that statin-treated dyslipidemia was associated with a lower risk of in-hospital mortality when compared to patients with no dyslipidemia. However, it remains unknown whether the effect was due to early or continuing statin treatment. Though it is important to describe in detail ST-segment change (ST-segment elevation, left bundle branch block, ST-segment depression, or T-wave inversion), details of ST-segment change were not evaluated in this study. Moreover, the usage rate of drug eluting stent (DES) was low in this study. In 2009–2010, many cardiologists recognized that the stent thrombosis rate of first-generation DES was increasing year by year [34–36]. The second generation of DES (such as the Everolimus-eluting stent) was released in Japan in 2010. Thus, many cardiologists may have avoided implanting DES to AMI patients due to these reasons.

Despite these limitations, we believe that our findings regarding acute situations in MI patients are accurate reflections within the contemporary DES and Troponin era in Tokyo.

## Conclusions

This metropolitan observational study revealed that STEMI and NSTEMI patients have different characteristics, managements, and prognoses. Compared with NSTEMI patient, those with STEMI generally have shorter door-to-balloon times, a higher proportion of PCI, more optimal medical therapy, but higher in-hospital mortality rates. In addition to the traditional negative independent predictors of in-hospital mortality such as age or Killip Classification, PCI performed within 24 h from onset and statin-treated dyslipidemia were positively associated with favorable in-hospital outcomes in STEMI patients. In contrast, for NSTEMI patients, PCI performed within 24 h from onset was proved to be the only positive factor to lower risk of in-hospital mortality. Moreover, in addition to the negative STEMI predictors, hemodialysis was found to be another an independent correlates of in-hospital mortality in NSTEMI patients.

## Appendix: Author contributions

Ayase Heart Hospital, Imun Tei; Bokutoh Metropolitan General Hospital, Ichiro Kubo; Disaster Medical Center, Yasuhiro Satoh; Edogawa Hospital, Yoji Oohira; Hakujikai Memorial Hospital, Kunio Tanaka; Higashiyamato Hospital, Masao Kawada; Ikegami General Hospital, Takao Machimura; IMS Katsushika Heart Center, Shigehiko Yoshida; Itabashi Chuo Medical Center, Hiroshi Ota; Japanese Red Cross Medical Center, Hiroshi Ikenouchi; Jikei University Daisan Hospital, Takahiro Shibata; Jikei University Hospital Katsushika Medical Center, Shingo Seki; Juntendo University Hospital, Hiroyuki Daita; Kanto Central Hospital, Akira Nozaki; Kanto Medical Center NTT EC, Satoshi Onishi; Kasai Shoikai Hospital, Akihiro Hata; Kawakita General Hospital, Yoichi Sugimura; Keio University Hospital, Tsutomu Yoshikawa; Kohsei Chuo Hospital, Akio Hirai; Kosei General Hospital, Masao Kawaguchi; Kyorin University Hospital, Hideo Yoshino; Mishuku Hospital, Akimi Uehata; Mitsui Memorial Hospital, Kazuhiro Hara; Musashino Red Cross Hospital, Tohru Obayashi; National Center for Global Health and Medicine, Hisao Hara; National Hospital Organization, Tokyo Medical Center, Yukihiko Momiyama; Nihon University Itabashi Hospital, Tadateru Takayama; Nihon University Nerima Hikarigaoka Hospital, Seiji Fukushima; Nippon Medical School Hospital, Keiji Tanaka; Nippon Medical School Tama-Nagayama Hospital, Hirotsugu Atarashi; Nishiarai Heart Center Hospital, Katsumi Saito; Nishitokyo Central General Hospital, Hiroyuki Suesada; Ogikubo Hospital, Yasuhiro Ishii; Ome Municipal General Hospital, Shigeo Shimizu; Saiseikai Central Hospital, Hideo Mitamura; Sakakibara Heart Institute, Tetsuya Sumiyoshi; Senpo Tokyo Takanawa Hospital, Masato Yamamoto; Showa General Hospital, Yuji Kira; Showa University Hospital, Youichi Kobayashi; Social Insurance Central General Hospital, Makoto Noda; St. Luke’s International Hospital, Yutaro Nishi; Surugadai Nihon University Hospital, Ken Nagao; Tama Nambu Chiiki Hospital, Tatenori Suzuki; Tama-Hokubu Medical Center, Satoshi Murasaki; Teikyo University Hospital, Takaaki Isshiki; The Cardiovascular Institute, Akira Koike; The Jikei University Hospital, Michihiro Yoshimura; The University of Tokyo Hospital, Ryozo Nagai; Tobu Chiiki Hospital, Takashi Tamura; Toho University Omori Medical Center, Nobuya Koyama; Toho University, Ohashi Medical Center, Masato Nakamura; Tokai University Hachioji-Hospital, Yoshinori Kobayashi; Tokyo Heart Center, Joji Hosokawa; Tokyo Kosei Nenkin Hospital, Seiji Ayabe; Tokyo Medical and Dental University, Mitsuaki Isobe; Tokyo Medical University, Hachioji Medical Center, Hiroshi Kobayashi; Tokyo Medical University Hospital, Akira Yamashina; Tokyo Metropolitan Geriatric Hospital, Kazumasa Harada; Tokyo Metropolitan Hiroo Hospital, Harumizu Sakurada; Tokyo Metropolitan Police Hospital, Tetsuro Shirai; Tokyo Metropolitan Tama Medical Center, Tetsuro Ueda; Tokyo Rinkai Hospital, Toru Kohno; Tokyo Women’s Medical University Hospital, Nobuhisa Hagiwara; Tokyo Women’s Medical University Medical Center East, Kuniaki Otsuka; Tokyo-kita Social Insurance Hospital, Yoshio Tsuruya; Toranomon Hospital, Sugao Ishiwata; Toshima Hospital, Takashi Shibui (67 hospitals).
